# A Review on Computer Vision-Based Methods for Human Action Recognition

**DOI:** 10.3390/jimaging6060046

**Published:** 2020-06-10

**Authors:** Mahmoud Al-Faris, John Chiverton, David Ndzi, Ahmed Isam Ahmed

**Affiliations:** 1 School of Energy & Electronic Engineering, Faculty of Technology, University of Portsmouth, Portsmouth PO1 3DJ, UK; john.chiverton@port.ac.uk (J.C.); ahmed.ahmed5@myport.ac.uk (A.I.A.); 2 School of Computing, Engineering and Physical Sciences, University of the West of Scotland, Paisley PA1 2BE, UK; David.Ndzi@uws.ac.uk

**Keywords:** human action recognition, hand-crafted feature, deep learning, feature representation

## Abstract

Human action recognition targets recognising different actions from a sequence of observations and different environmental conditions. A wide different applications is applicable to vision based action recognition research. This can include video surveillance, tracking, health care, and human–computer interaction. However, accurate and effective vision based recognition systems continue to be a big challenging area of research in the field of computer vision. This review introduces the most recent human action recognition systems and provides the advances of state-of-the-art methods. To this end, the direction of this research is sorted out from hand-crafted representation based methods including holistic and local representation methods with various sources of data, to a deep learning technology including discriminative and generative models and multi-modality based methods. Next, the most common datasets of human action recognition are presented. This review introduces several analyses, comparisons and recommendations that help to find out the direction of future research.

## 1. Introduction

Human Action Recognition (HAR) has a wide-range of potential applications. Its target is to recognise the actions of a person from either sensors or visual data. HAR approaches can be categorised into visual sensor-based, non-visual sensor-based and multi-modal categories [1,2]. The main difference between visual and other categories is the form of the sensed data. The visual data are captured in the form of 2D/3D images or video whilst others capture the data in the form of a 1D signal [2]. Over the last few years, wearable devices such as smart-phones, smart-watches, and fitness wristbands have been developed. These have small non-visual based sensors and are equipped with computing power and communication capability. They are also relatively low cost which has helped to open up new opportunities with ubiquitous applications. These include health monitoring, recuperative training and disease prevention, see, e.g., [3].

At the same time, visual sensor-based methods of human action recognition are one of the most prevalent and topical areas in the computer vision research community. Applications have included human–computer interaction, intelligent video surveillance, ambient assisted living, human–robot interaction, entertainment and content-based video search. In each one of those applications, the recognition system is trained to distinguish actions carried out in a scene. It may also perform some decisions or further processing based on that inference.

It can be stated that wearable devices have several limitations such as in most cases they need to be worn and to operate constantly. This might be a significant issue for real applications that may require readiness and deployability. In turn, requiring specific technical requirements related to e.g., battery life, size and performance of the sensor, see, e.g., [4]. In addition, they might not be suitable or efficient to employ in e.g., crowd applications or other related scenarios. These limitations are not applicable to computer-vision based HAR. Computer vision based HAR can be applied to most of application scenarios without these technical requirements or limitations.

From about 1980, researchers have presented different studies on action recognition based on images and/or video data [5,6]. In many instances, researchers have been following or drawing inspiration from elements of the operating principles of the human vision system. The human vision system receives visual information about an object especially with respect to movement and shape and how it changes with time. Observations are fed to a perception system for recognition processes. These biophysical processes of the human recognition system have been investigated by many researchers to achieve similar performance in the form of computer vision systems. However, several challenges such as environmental complexities, scale variations, non-rigid shapes, background clutter, viewpoint variations and occlusions make computer vision systems unable to fully realise many elementary aspects of a human vision system.

Action recognition systems can be categorised into different four categorises according to the complexity of human action. This can include: primitive [7], single person [8], interaction [9], and group [10] actions recognition. Primitive action indicates basic movement of human body parts—for example, “lifting a hand” and “bending”. Single person actions indicate a set of primitive actions of a single person such as “running” and “jumping”. Interaction indicates actions involve humans and objects, such as “carrying a box” and “playing a guitar”. Group actions refer to actions occurring in a group of people such as a “procession”, “meeting”, and “group walking”.

In general, computer vision methods based HAR can be classified into two categories in terms of a comprehensive investigation of the literature: (a) Traditional hand-crafted feature based methods followed by a trainable classifier for action recognition. In addition, (b) deep learning based approaches are able to learn features automatically from raw data and are commonly followed by a trainable classifier for action recognition [11,12].

Many important survey and review papers have been published on human action recognition and related techniques. However, usually, published reviews go out-of-date. For this reason, writing an updated review on human action recognition is significantly required although it is considered hard work and a challenging task. In this review, discussions, analysis and comparisons of state-of-the-art methods are provided for vision based human action recognition. Handcrafted based methods and deep learning based methods are introduced along with popular benchmark datasets and significant applications. This paper also considered different designs of recognition models including: hybrid, modalities-based and view-invariant based. A brief detail of different architectures is introduced for vision-based action recognition models. Recent research works are presented and explained to help researchers to follow the path for possible future works.

The structure of this review starts at low level based methods for action recognition. This is followed by description of some of the important details of feature descriptor based techniques. A number of improvements that can be achieved in these aspects are identified. These are also transferable with respect to the performance of action recognition systems in general. Thereafter, it reviews higher level feature representation based methods. It explains the widespread feature descriptor based techniques with respect to different aspects. The paper then covers the mainstream research that has resulted in the developments of the widely known deep learning based models and their relation to action recognition systems.

## 2. Popular Challenges in Action Recognition Models

Initially, it might be useful to highlight some of the most popular challenges in action recognition based methods.

### 2.1. Selection of Training and Testing Data

The type of data can strongly affect the efficiency of a recognition model. Three types of data are usually used for action recognition. These are RGB, depth, or skeleton information, each of which can have advantages and disadvantages. For instance, significant texture information can be provided from an RGB input. This might be considered to be closely related to the visual information that humans typically process. On the other hand, a lot of variations can occur in the appearance information that depend on e.g., lighting conditions. In contrast to RGB, depth map information is invariant to illumination changes. This makes it easier to detect foreground objects from the background scene. In addition, a depth map provides 3D characteristics about the captured scene. However, depth map information also commonly has some defects. For instance noisy measurements are sometimes a problem need to be purified and refined. Another input type is skeleton information. Skeletons can be obtained using different approaches; see, e.g., [13,14,15,16]. Skeleton can be obtained from RGB or more commonly depth information. However, this type of information is often captured or computed imperfectly especially in an occluded or noisy environment. In this work, the complementary information available in the RGB and depth map data are exploited directly for action recognition.

### 2.2. Variation in Viewpoint

Most methods assume that actions are performed from a fixed viewpoint. However, in a real case, the location and posture of the person vary considerably based on the viewpoint where the action is captured from. In addition, a variation in motion patterns are also appeared in each different view which makes recognition of an action more difficult. Training a classifier using multiple camera information is a way used by [17] to tackle this issue. View-invariant representation was also obtained by modeling a 3D body posture for action recognition such in [18]. Researchers try to to utilise view-invariant features space using Fourier transform and cylindrical coordinate systems [19]. However, researchers [20,21] have reported that most multi-view datasets involve uniform or fixed background. Therefore, in order to evaluate the performance of various methods, it would be necessary to validate those using actions recorded in real-world settings.

### 2.3. Occlusion

An action required to be recognised should be clearly visible in the video sequences. This is not true in the real case, especially in a normal surveillance video. Occlusion can be presented by the person itself or by any other objects in the field. This can make body parts performing an action invisible which can cause a big issue for the research community. Volumetric analysis and representation [22] of an action can tackle self-occlusion issues and helps to match and classify the action. Considering body parts separately is a feasible way to handle occlusions. This can be performed using Pose-based constraints [23] and Probabilistic-based methods [24,25]. The multiple camera setup method is another approach that is used by researchers to handle occlusion problems [26].

### 2.4. Features Modelling for Action Recognition

In general, two popular methods are found to be considered for designing features for action recognition. One can use feature design based application methods which lead to the utilisation of the hand-crafted features. Another way is to automatically capture features from input data. This can be achieved using deep learning techniques which have often shown competitive performance in comparison to hand-crafted feature based methods [27].

### 2.5. Cluttered Background

Cluttered background is a case that formed a distraction introducing ambiguous information in the video of an action [28]. Different vision-based methods are affected by this issue such as an optical flow algorithm that is used to calculate motion information but with unwanted background motion (due to cluttered background) along with the required motion. In addition, this issue has a great influence on colour-based and region-based segmentation approaches as these methods require uniform background to achieve high quality segmentation. In order to handle and avoid the issues introduced, many research works assumed a static background or an approach to deal with the videos prior to processing [20,29].

### 2.6. Feature Design Techniques

Different levels of features can be used for action recognition. Some researchers such as [30,31,32] proposed to employ the input as a whole referred to here as holistic methods. Other researchers such as [33,34,35,36] considered salient points of interest from input data with what are known as local feature based methods.

Motion is an important suorce of information that needs to be considered for action recognition. Different techniques have been proposed to model motion information in the feature computation step. This has included optical flow for low level feature displacements and trajectories across multiple frames which can then be fed to classifiers or to further feature extraction processes. Some other research has included motion information in the classification step with models such as: Hidden Markov Models [37]; Conditional Random Fields [38]; Recurrent Neural Network [39]; Long-Short Term Memory; and 3D Convolution Neural Network [40]. All of these are able to model sequential information by design.

In such systems, an efficient feature set is able to reduce the burden for improving the recognition. An overview is now provided of selected state-of-the-art methods with respect to all aforementioned challenges and approaches mentioned above. In the following, action recognition systems are partitioned based on hand-crafted features in addition to those based on different deep learning techniques.

## 3. Applications of Action Recognition Models

During the last decade, many researchers have paid attention to the action recognition field with a significant evolution of the number of publications. This section highlights state-of-the-art applications that consider human action recognition methodologies to assist humans. Different applications of the current action recognition approaches are discussed including: smart homes and assisted living, healthcare monitoring, security and surveillance, and human–robot interaction [41,42].

### 3.1. Surveillance and Assisted Living

Different modern technologies have provided a wide range of improvements in the performance of independent assisted living systems. This comes true using action recognition techniques to monitor and assist occupants. For example, a smart home system proposed by [43] used machine learning and features extraction techniques to analyse the activity patterns of an occupant to introduce automation policies based on the identified patterns to support the occupants. Another smart system has been introduced by [44] for human behaviour monitoring and support (HBMS). This was achieved by observing an occupant’s daily living activities using the Human Cognitive Modeling Language (HCM-L). Then, the HBMS control engine is applied to assist individuals in a smart way. On the other hand, vision-based technologies are introduced in different security applications such as the surveillance system that introduced by [45]. This system has the ability to recognise human behaviours such as fighting and vandalism events that may occur in a public district using one or several camera views [46]. Multiple camera views were used by [47] to detect and predict suspicious and aggressive behaviours in real time and in a crowded environment.

### 3.2. Healthcare Monitoring

The development of medical research and technology remarkably improved the quality of patients’ life. However, higher demands of medical personnel made researchers try different technologies to improve healthcare monitoring methods that may be essential in emergency situations. Basically, one or more factors can be involved in the design of healthcare monitoring systems. This can include fall detection, human tracking, security alarm and cognitive assistance components. In [48], a vision-based system was proposed for healthcare purposes. It used Convolutional Neural Networks to detect person falling. Optical flow sequences were used as input to the networks followed by a three training phases. Fall detection system for home surveillance was proposed by [49]. A surveillance video was used to detect the fall. Background subtraction was used to detect the moving object and segmented within a bounding box. Few rules were used with the transitions of a finite state machine (FSM) to detect the fall based on the measures of the extracted bounding box. An intelligent monitoring system was proposed by [50] to monitor the “elopement” events of dementia units and to automatically alert the caregivers. Audio and video daily activities were collected and detected using an HMM-based algorithm.

### 3.3. Entertainment and Games

In the recent years, gaming industries have developed a new generation of games based on the full body of a gamer such as dance and sports games. RGB-D sensors (see, e.g., [51]) are used in this kind of games to improve the perception of human actions. A rich information of an entire scene is provided by these sensors to facilitate action recognition tasks [52,53].

### 3.4. Human–Robot Interaction

Human–robot interaction is considerably adapted in home and industry environments. An interaction is achieved to perform a specific task such as “Passing a cup” or “locating an object”. A vision-based method is one of the effective communication ways between human and robots [54,55].

### 3.5. Video Retrieval

Most search engines use the associated information to manage video data. Text data such as tag, description, title and keywords is one piece of information that can be used for such purposes [56]. However, one piece of information can be incorrect, which results in unsuccessful video retrieval. An alternative approach was proposed by [57] for video retrieval by analysing human actions in videos. The designed framework computed the similarity between action observations to then be used to retrieve videos of children with autism in a classroom setting.

### 3.6. Autonomous Driving Vehicles

An automated driving system is aimed to ensure safety, security, and comfort. One of the most important components of this system is action prediction and recognition algorithms [55,58]. These methods can analyse human action and motion information in a short period of time that helps to avoid critical issues such as collision.

## 4. Hand-Crafted Feature Representation for Action Recognition

We will start by demonstrating some classical human action recognition based methods based on hand-crafted features. Classical image classification based methods usually consist of three consecutive steps: features extraction, local descriptor computation and classification. Similar steps have been employed more generally for image and video classification as well as human action recognition.

### 4.1. Holistic Feature Representation Based Methods

Holistic feature representation based methods treat Regions Of Interest (ROI)s as a whole in which all pixels are exploited to compute the descriptors. In general, holistic based methods consist of two steps for action recognition which are person detection and descriptor computation. Holistic methods consider a global structure of the human body to represent an action, where it is not necessary to localise body parts. The key idea is that discriminative global information can be represented from a region of interest which can then be used for action characterisation. Holistic methods can be efficient and effective in addition to simple to compute due to the use of global information only. This makes this kind of method important for videos which might contain background clutter, camera motion, and occlusions.

In general, holistic methods can be classified into two categories based on the information that is used for the recognition problem:

- Recognition based on shape information such as shape masks and the silhouette of the person;
- Recognition based on shape and global motion information.

#### 4.1.1. Shape Information Based Methods

Holistic based approaches are often based on information from the silhouettes, edges, optical flow, etc. Such methods are sensitive to noise, background clutter, and variations in occlusion and view-points e.g., see [59]. Silhouette information provides shape information about the foreground in the image. Different techniques can be employed to compute silhouette information from the background scene. One simple technique is background subtraction that can be used with high confidence when the camera is static. On the other hand, some research such as in [60] has utilised human tracker and camera motion estimation to obtain silhouette information and to cope with the drawbacks of camera motion. Shape information can be utilised in the time domain to help to consider the evolution of the silhouette over time. Differences in the binary silhouettes have considered by [61]. These were accumulated in the spatial and temporal domains to construct a Motion Energy Image (MEI) and a Motion History Image (MHI), respectively. These depict an action with a single template. MEI is a binary template that indicates regions of movement. MHI indicates regions of motion where more recent motion regions have higher weight. Three-dimensional (3D) shape information was used by [31] for action recognition by stacking 2D silhouette information into a space-time volume. For invariant representations to geometrical transformations such as scaling and translation, an extended Random transform was proposed by [62]. This was applied to binary silhouette information for action recognition. Contours of MEI templates were exploited by [63]. A descriptor was obtained which was found to be invariant to scale changes and translations.

A lot of research has utilised shape and silhouette information to represent the human body for human action recognition. In [30,64], shape masks of different images were used to introduce MEI and MHI based temporal templates for action recognition.

It has been observed that some actions can be represented by key poses. This was proposed by [65] where a method was described to detect forehand and backhand tennis strokes by matching edge information to labelled key postures together with annotated joints. These were then tracked between the key consecutive frames based on the silhouette information.

A number of significant methods are presented by [66] to describe space-time shapes based on silhouette information for action recognition. Background subtraction was used to extract the silhouette of a person. The Poisson equation was then used to obtain saliency, dynamics and shape structure features. A high dimensional feature vector was introduced to describe sequences of 10 frames in length. This was matched to shapes of test sequences at the end.

Space-time shapes were also used by [67] where contour information was obtained using background subtraction. Then, a set of characteristic points (saddles, valleys, ridges, peaks and pits) were used to represent actions on the surface of the shape. The space-time shapes were matched to recognise actions using point-to-point correspondences.

In [68], a set of silhouette exemplars were used for matching against frames in action sequences. A vector was formed of the minimum matching distance between each exemplar and any frame of the sequence. A Bayes classifier was employed to learn action classes with two different scenarios: first, silhouette information; second, edge information.

A foreground shape based motion information model was presented by [69] to represent motion from a group of consecutive frames of an action sequence. A motion context descriptor was introduced over a region with the use of a polar search grid, where each cell was represented with a SIFT descriptor [70]. The final descriptor was created by summing up the entire groups of a sequence. After that, three different approaches were used to recognise actions which were Probabilistic Latent Semantic Analysis (pLSA) [71], w3-pLSA (pLSA extension) and Support Vector Machine (SVM).

Colour and location information based segmentation has been used by [72] to automatically over-segment event video. Then, optical flow and volumetric features were used to match over-segmented video against a set of training events such as picking up a dropped object or waving in a crowd.

It is obvious from the aforementioned approaches that silhouette information can provide strong cues for the human action recognition problem. However, significant challenges arise in the presence of clutter, occlusion and camera motion. In addition, silhouette information can describe some types of actions by showing characteristics of the outer contours of a person. However, other actions that include, e.g., self-occlusion, may not easily be recognised from silhouette information alone. Therefore, the motion and shape information is further enhanced with the use of local feature representations discussed shortly.

##### RGB-D Information Based Shape Models

A new era can be considered to have begun when low cost RGB-D sensors were produced. These simultaneously provide appearance and spatial 3D information. Such devices (e.g., Microsoft Kinect, Asus Xtion) have the ability to work in real time. By adding the depth-map feature, the device is able to provide information about the distance of each pixel to the sensor in a range from 0.5 m to 7 m. These have played a key role in the enhancement of object detection and segmentation algorithms. RGB-D sequences based methods improve recognition performance with a low time complexity. However, depth and skeleton representation based methods of action recognition remain only applicable over a limited range and specific environmental conditions.

As a result, many RGB holistic approaches have been extended to the RGB-D scenario to utilise depth-map characteristics. A 3D-MHI has proposed by [73] for action recognition. This was performed by extending the traditional MHI to use depth information. In [74], the depth silhouette was sampled into a representative set of 3D points and used to introduce the shape of salient regions. The key idea was to project the depth map onto three orthogonal Cartesian planes and use the points along each plane to recognise the actions. A useful technique was used by [75] where the depth maps were projected onto three orthogonal Cartesian planes to produce Depth Motion Maps (DMM) by combining through summation the stacked motion energy of each of the projected maps. DMMs can express the variation of a subject’s motions during the performance of an activity. In [76], DMMs were used for activity recognition together with an $l_{2}$-regularised collaborative representation classifier with a distance-weighted Tikhonov matrix was also used. DMMs was used by [77] with Local Binary Patterns (LBP)s to utilise motion cues. Two fusion levels were also considered including feature-fusion level and decision-fusion level. The DMM based results showed reasonable human activity recognition performance.

Different levels of the same data sequence have been used with DMM computations to create a hierarchical DMMs in [78]. An LBP based descriptor was used to characterise local rotation invariant texture information. Then, a Fisher kernel was employed to create patch descriptors. These were fed into a kernel-based extreme learning machine classifier. A similar approach was followed by [79]. A Histogram of Oriented Gradients (HOG)s descriptor was used along with kernel entropy component analysis for dimensionality reduction. Finally, a linear support vector machine was used in the classification. For both hierarchical DMM based approaches, the results demonstrated a significant performance improvement.

A 4D space-time grid has introduced by [80] that extended the work by [31]. This has done by dividing space and time dimensions into multiple cells. These were used to obtain Space Time Occupancy Patter (STOP) feature vectors for action recognition. In [81], a 4D Histogram Of Surface Normal Orientations (HON4D) was proposed to describe video for action recognition after computing the normal vectors for each frame. The features of the surface normal were captured in the 4D space of spatial, depth and time dimensions.

The rich characteristics of the depth information can help make people detection and segmentation tasks easier and less challenging which in turn improves holistic approaches, making them more robust with RGB-D images. However, some drawbacks of holistic methods include their sensitivity to occlusions and noise in the depth maps. Therefore, a good representation can be presented by combining motion and shape information which in turn may improve the recognition rate of the system.

#### 4.1.2. Hybrid Methods Based on Shape and Global Motion Information

The work by [82] is a good example of shape and motion feature based tracking and action recognition. The authors assumed that the movements of body parts were restricted to regions around the torso. Subjects were bounded with rectangular boxes where the centroids were selected as the feature for tracking. The velocity of the centroids was considered, utilising body motion features to cope with occlusions between multiple subjects. Periodic actions such as walking were detected with a nearest centroid algorithm calculated across spatio-temporal templates and reference templates. This approach, however, only utilised motion information which can be improved by considering other features such as texture, color, and shape.

Another method which used motion information was proposed by [83] based on optical flow to track soccer players and to recognise simple actions in video. A person was tracked and stabilised. Then, a descriptor was computed over the motion information and spatio-temporal cross-correlation was used for matching with a database. This approach was tested on sequences from ballet, tennis and football datasets, and it achieved impressive results on low resolution video. However, their types of systems may depend on several conditions such as position of the region of interest in the frame, spatial resolution and relative motion with respect to the camera. In addition, the model is based on a global representation which can be affected by occlusions between multiple objects and a noisy environment in the background.

Flow motion has also been used by [84] for action recognition. A flow descriptor was employed to select low level features in the form of a space-time overlapped grid. Then, mid level features were selected using the AdaBoost algorithm.

A space-time template based method was introduced by [85] for action recognition. It was based on the maximum average correlation height filter. A spatio-temporal regularity flow was used to capture spatio-temporal information and to train a Maximum Average Correlation Height (MACH) filter. Experiments on a number of datasets including the KTH dataset demonstrated action recognition and facial expression recognition.

Volumetric feature based action recognition was proposed by [86] where Viola–Jones features were computed over a video’s optical flow. A discriminative set of features were obtained by direct forward feature selection which employed a sliding window approach to recognise the actions. The model was trained and tested on real videos with actions that included sit down, stand up, close laptop and grab a cup actions.

Shape information was used by [87] to track an ice hockey player and to recognise actions. Histograms of Oriented Gradients (HOG)s were used to describe each single frame. Principal Component Analysis (PCA) was then used for dimensionality reduction. At the end, a Hidden Markov Model (HMM) was employed to recognise actions.

A new technique was proposed to utilise a hybrid representation by combining optical flow and appearance information by [88]. They exploited the optical flow information and Gabor filter features for action recognition. Both kinds of features were extracted from each single frame and then concatenated. They used different lengths of snippets of frames to highlight how many frames were required for recognising an action. The Weizmann and KTH datasets were used for evaluation schemes.

Motion and shape information based action recognition was also used by [89] where a multiple instance learning based approach was employed to learn different features from a bag of instances. This included foreground information, Motion History Image (MHI) and HOGs. Simple actions in crowded events in addition to shopping mall data were used to evaluate the proposed method. The experiments showed that the use of multiple types of features resulted in better performance in comparison with a single type of feature.

These holistic based methods have provided some reasonable levels of performance for action recognition. However, they are not view invariant. Different models would be needed for particular views. Large amounts of multiple view data would also be needed for training. Some body parts might be unseen across frames due to occlusions. Second, they are not invariant to time. The same action performed over different time periods would present quite differently. In addition, it is worth mentioning that the accuracy of holistic approaches is highly dependent on the detection and segmentation pre-processing. This work also includes local representation based methods to benefit from localised information. The next section presents a review of the local representation based methods for human action recognition.

### 4.2. Local Feature Representations Based Methods

Local feature based methods tend to capture characteristic features locally within a frame without a need for human detection or segmentation which can be quite a challenge for RGB based video. Local feature based methods have been successfully employed in many recognition system applications such as action recognition [90], object recognition [91] and scene recognition [92]. Local capture based methods can capture important characteristics of shape and motion information for a local region in a video. The main advantage of these methods is the autonomous representation of events in terms of changes across space-time and scale. Furthermore, with appropriate machine learning, it is often possible, given sufficient data, to capture the important characteristics of the local features of interest. If appropriately achieved, then it can be possible to separate these features from features computed from a cluttered background or even multiple movements or objects in a scene. In the following section, space-time feature detectors, feature trajectories and local descriptor based methods are discussed. In addition, the incorporation in action localisation and recognition in videos will be considered.

In general, local feature based methods consist of two steps: detecting a point of interest (POI) and descriptor computation. In image processing, interest points refer to points that have local variation of image intensities. Interest point detectors usually capture local characteristics. This can be in terms of space-time and scale in videos by maximising specific saliency functions.

Some research that can be highlighted has focused on feature detectors such as [33] who proposed to extend the Harris corner detector to a Harris3D detector to include both space and time. A different feature detector which employed spatial Gaussian kernels and temporal Gabor filters was proposed by [93]. This considered salient motion features to represent different regions in videos. Another detector proposed by [94] involved computing entropy characteristics in a cylindrical neighborhood around specific space-time positions. An extension of the Hessian saliency detector, Hessian3D, was proposed by [95] to consider spatio-temporal features. This used the determinant of the 3D Hessian matrix. Salient features were detected by [96] using a global information based method.

A wider experimental evaluation was introduced by [97]. They proposed to exploit different interest point detectors applied to publicly available action recognition datasets including KTH [98], UCF sports [85], and Hollywwod2 [99]. The results showed the robustness of dense sampling method, where interest points were sampled in equal segments in the space and time domains. It was found that the Harris3D detector achieved some of the best performance in some of the included experiments.

While local interest points are detected, local representation based methods can then be employed to compute one of the different descriptors over a given region. Different descriptors have been proposed in a lot of research such in [34] where Histogram of Oriented Gradients (HOG) [100] and Histogram of Oriented Optical Flow (HOOF) [101] descriptors were used. The authors introduced a different way to characterise local motion and appearance information. They combined HOG and HOOF based approaches on the space-time neighbourhood of the detected points of interest. For each cell of a grid of cells, four bins of HOG and five bins of HOOF were considered. Normalised and concatenation were used to form a HOG and HOOF combined descriptor. Moreover, different local descriptors based on gradient, brightness, and optical flow information were included by [93]. PCA was also used for dimensionality reduction. The authors explored different scenarios which included simple concatenation, grid of local histograms and a single global histogram. The experimental results determined that concatenated gradient information achieved the best performance.

A 3D version of the Histogram of Oriented Gradients (HOG3D) has introduced by [102] as an extension of the HOG descriptor by [100]. A space-time grid was constructed around each detected Point Of Interest (POI). A histogram descriptor was then computed and normalised over each of the cells. The final descriptor was then formed by concatenating the histograms.

In [103], the authors proposed to extend the Scale-Invariant Feature Transform (SIFT) descriptor originally proposed by [70]. Spatio-temporal gradients were computed over a set of randomly sampled positions. A Gaussian weight was used to weight each pixel in the neighbourhood with votes into an N×N×N grid of histograms of oriented gradients. To achieve orientation quantization, the gradients were represented in spherical coordinates that were divided into 8×4 histograms.

An extended Speeded-Up Robust Features (SURF) descriptor originally proposed by [104] was investigated by [95]. Application to videos was considered by utilising spatio-temporal interest points which were spatially and temporally scale invariant. The patches were divided into a grid within local N×N×N histograms. Then, each cell was represented by a vector of Haar wavelet sampled responses. The experimental results showed the good performance of the proposed detector in comparison with other detectors.

#### RGB-D Information Based Local Features

There has also been research that includes depth map data based local feature methods. These follow many of the same or similar steps as for RGB video. For instance, at the gross level, finding salient points of interest and then computing the descriptor. In [105], the authors proposed a Histogram of Oriented Principal Components (HOPC) descriptor. This captured the characteristics around each point of interest within a 3D cloud space. The descriptor was formed by concatenating projected Eigenvectors. These resulted from Principal Component Analysis on the space-time volume around the points of interest. The HOPC descriptor was found to be view invariant. Video was also treated in [106] as a space-time volume of depth values. A Comparative Coding Descriptor (CCD) was then used to encode space-time relations of points of interest. Set of cuboids were used to construct a series of codes that characterised the descriptor. In [107], a descriptor called Local Occupancy Pattern (LOP) was presented. This was used to describe the appearance information of sub-regions of depth images by which was utilised to characterise object-interaction actions. In another work by [108], a Random Occupancy Pattern (ROP) was introduced to deal with depth sequences as a space-time volume. The descriptor was defined by a sum of the pixel values in a sub-volume. Since several sub-volumes had different sizes and locations, a random sampling based method was used to effectively recognise the sub volumes. Overall, local feature based methods are commonly used with different inputs. These can include skeletons where joints have been a particular focus for detector, RGB where a detector have been used to detect POIs on an RGB frame, or similarity for the depth.

### 4.3. Trajectories Based Methods

Many researchers have claimed that the spatial domain in video has different characteristics from the temporal domain. Thus, points of interest should not be detected in a 3D spatio-temporal space. Consequently, a lot of research such as [36,101,109,110,111] has included tracking of detected points of interest across the temporal domain. Then, the volume of the trajectory points are often used to compute the descriptors for video representation.

Detecting points of interest in video and forming trajectories through the temporal domain has been used by many researchers. For instance, the Kanade–Lucas–Tomasi (KLT) tracker [112] was used in [109] to track Harris3D interest points [33]. These formed feature trajectories which were then represented as sequences of log polar quantised velocities. The KLT tracker has also been used by [36], where trajectories were clustered and used to compute affine transformation matrix to represent the trajectories. In [70,110], SIFT descriptors were matched between two consecutive frames for trajectory based feature extraction. Unique-match points were exploited whist others were discarded.

Dense sampling based interest point extraction achieved better performance in action recognition by [97]. Dense trajectories were later used by [101] who sampled dense points of interest on a grid. Dense optical flow was then used to track POIs through time. Trajectories were formed by concatenating points from subsequent frames. Moreover, to exploit motion information, different descriptors (HOG, HOOF, Motion Boundary Histogram (MBH)) were computed within a space-time volume around the trajectory. Finally, the method was evaluated with publicly available action datasets including: KTH, YouTube, Hollywood2, and UCF sports. Competitive performance was achieved in comparison to the state-of-the-art approaches. Different extensions of dense trajectory based methods have been proposed by many researchers such as [113,114,115,116,117,118].

Local descriptor based methods often follow similar steps in comparison to POI detection. Early research extracted descriptors from cuboids which were formed around the point of interest in space-time domains, see, e.g., [33,93]. However, the same process can be followed to utilise trajectories. Most popular local descriptor based approaches have exploited cuboids or trajectories as explained below.

A number of different descriptors were introduced by [119] to capture appearance and motion features from video. A comparison between single and multi scale higher order derivatives, histograms of optical flow, and histograms of spatio-temporal gradients was developed. The local neighbourhood of the detected interest points was described by computing histograms of optical flow and gradient components for each cell of a N×N×N grid. Thereafter, PCA was applied to the concatenation of optical flow and gradient component vectors to exploit the most significant eigenvalues as descriptors. The experiments showed the usefulness and applicability of the histograms of optical flow and spatial-temporal gradient based descriptors.

The Histograms of Optical Flow (HOOF) descriptor was proposed by [34] to identify local motion information. Spatio-temporal neighbourhoods were defined around detected POIs and optical flow was computed between consecutive frames.

Another robust descriptor, which also benefited from optical flow, was presented by [120] to extract local motion information called the Motion Boundary Histogram (MBH) descriptor. This descriptor follows the HOG descriptor in binning the orientation information of spatial derivatives into histograms. These descriptors can be employed with trajectory information as was done by [121]. A spatio-temporal volume was formed around each trajectory and divided into multiple cells. Each cell was represented by a combination of HOG, HOOF and MBH descriptors. Some other research that used trajectories for action recognition can be found such as [122,123,124].

### 4.4. Other Feature Representations Based Methods

A different representation method has been employed in computer vision tasks called Bag of Words (BOW) also referred to as a bag of visual models; see, e.g., [125]. The key idea of this approach is to represent image data as a normalised histogram called code words. The visual words (code words) can be constructed during the learning process by clustering similar patches of an image that can be described by a common feature descriptor. In this way, some techniques will result in similar histograms for similar images. These can be fed into a classification step. BOW based methods have been used in a lot of research for action recognition such as [28,93,126,127].

Another popular feature representation technique is the Fisher vector descriptor which can be considered as a global descriptor. This technique determines the best calibration for a generative model to better model the distribution of extracted local features. The descriptor is formed using the gradient of a given sample’s likelihood with respect to the parameters of the distribution. It is estimated from the training set and scaled by the inverse square root of the Fisher information matrix. A Fisher vector descriptor was first presented by [128] for image classification. For more details about Fisher vector based image classification and action recognition tasks, please see [129,130].

More comprehensive details of action recognition, motion analysis, and body tracking can be also found in [131,132,133,134,135]. Some state-of-the-art works that used traditional hand-crafted representation based methods are presented and compared in Table 1.

It is worth pointing out that a variety of higher-level representations techniques have been proposed to capture discriminative information for complex action recognition. Deep learning is an important technique that has demonstrated effective capability for producing higher-level representations with significant performance improvement. Deep learning based models have the ability to process input data from a low level and to convert it into a mid or high-level feature representation. Consequently, the next section presents a good review of deep learning based models that have been used for human action recognition.

## 5. Deep Learning Techniques Based Models

Recent research studies have shown that hand-crafted feature based methods are not suitable for all types of datasets. Consequently, a new relatively and important class of machine learning technique referred to as deep learning has been established. Multiple levels of feature representations can be learnt that can make sense of different data such as speech, image and text. Such methods are capable of automatically processing raw image and video data for feature extraction, description, and classification. Trainable filters and multiple layer based models are often employed in these methods for action recognition and representation.

This section presents descriptions of some important deep learning models that have been used for human action recognition. However, it is very difficult to train a deep learning model from scratch with limited data. Thus, models are often limited to appearance based data or some described representation. Deep learning based models can be classified into three categories which are: generative models e.g., Deep Belief Networks (DBNs), Deep Boltzmann machines (DBMs), Restricted Boltzmann Machines (RBMs), and regularized auto-encoders; supervised models e.g., Deep Neural Networks (DNNs), Recurrent Neural Networks (RNNs), and Convolutional Neural Networks (CNNs); and hybrid models. However, hybrid models are not discussed in this work.

### 5.1. Unsupervised (Generative) Models

The key idea of deep learning based generative models is that they do not need target labels for the learning process. Such models are appropriate when labelled data are scarce or unavailable. The evolutionary of deep learning models can be traced back [158] where a Deep Belief Network (DBN) was presented with a training algorithm based on Restricted Boltzmann Machines (RBMs) [159]. This was followed by a dimensional reduction technique by [160]. The parameters were learnt with an unsupervised training process which were then fine-tuned in a supervised approach using back-propagation.

This inspired great interest in deep learning models particularly on different applications such as human action recognition, image classification, object recognition, and speech recognition. Unsupervised learning based methods have been proposed by, e.g., [161], to automatically learn features from video data for action recognition. An independent subspace analysis algorithm was used to learn space-time features and combined with convolution and stacking based deep learning techniques for action representation.

In [162], the researchers proposed to train DBNs with RBMs for human action recognition. The experimental results on two public datasets demonstrated the impressive performance of the proposed method over hand-crafted feature based approaches.

An unsupervised deep learning based model was proposed by [163] to continuously learn from unlabelled video streams. In addition, DBNs based methods were used by [164] to learn features from an unconstrained video stream for human action recognition.

Generative or unsupervised learning based models have played a substantial role in inspiring researchers’ interest in the deep learning field. Nevertheless, the great development of the Convolution Neural Networks (CNNs) based supervised learning methods for object recognition has somewhat obscured the unsupervised learning based approaches; see, e.g., [165].

### 5.2. Supervised (Descriminative) Models

In line with the recent literature surveys for human action recognition, the most common technique used in supervised learning based models is Convolution Neural Networks (CNN)s. These were first proposed by [166]. CNNs can be considered to be a type of the deep learning model which has shown great performance in various recognition tasks such as pattern recognition, digit classification, image classification, and human action recognition see, e.g., [165,167]. The efficient utilisation of CNNs in image classification [165] opened a new era to employ deep learning based methods for human action recognition. The key advantage of CNNs is their ability to learn straight from the raw data such as RGB or depth map data. Consequently, it is possible to obtain discriminative features which can effectively describe the data and thus make the recognition process easier. Since this approach is susceptible to overfitting, one should be careful in the training process. CNN includes regularisation and has a significant requirement for a large amount of labeled data. These can help to prevent overfitting. Recently, it was shown that deep learning based methods outperform many state-of-the-art handcrafted features for image classification; see, e.g., [27,165,168].

Convolution Neural Networks (CNN)s have a hierarchical structure with multiple hidden layers to help translate a data sample into a set of categories. Such models consist of a number of different types of layers such as convolutional layers, pooling layers and fully connected layers. The temporal domain is introduced as an additional dimension in the case of videos. Since CNNs were originally designed for static image processing, it was not initially clear on how to incorporate motion information. Therefore, most research at that time used CNNs on still images to model appearance information for action recognition [165]. Thereafter, different ways were proposed to utilise motion information for action recognition. An extension was presented by [169] where stacked video frames were used as an input to a CNN for action recognition from video. However, the experimental results were worse than hand-crafted feature based approaches. An investigation made by [32] about this issue and developed the idea of having separate spatial and temporal CNN streams for action recognition.

**Figure 1:**
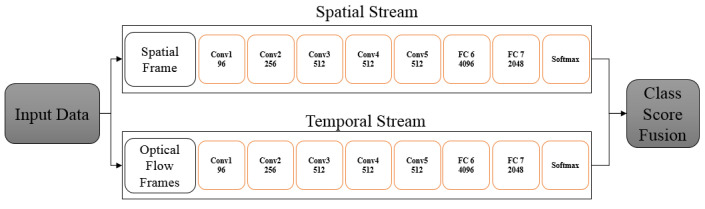
Illustration of the spatio-temporal CNN streams as used by [32]. Here, the input data are split into two streams, one for the individual apperance based raw frames. The other for the temporal information corresponding to an optical flow stream. The two streams are fused at the end with class score fusion.

Figure 1 illustrates the spatio-temporal CNN streams similar to [32] where the two streams are implemented as independent CNNs. One stream was the spatial stream which recognised actions from static images. The other stream was the temporal stream which recognised actions from stacked video frames based on motion information of dense optical flow. The output of the two streams was combined using a late fusion technique. The experiments showed improved performance for this method compared to hand-crafted feature based approaches. However, this type of architecture has additional hardware requirements to be suitable for a variety of applications.

A lot of research on action recognition is based on works that have previously achieved relatively good performance in image classification problems. Recent works extended what was implemented in two dimensions to 3D to include the temporal domain. Most CNN models proposed for action recognition have been limited to deal with 2D input data. Nonetheless, some applications may include 3D data that requires a specialised deep learning model. To this end, 3D Convolution Neural Networks (3D-CNNs) based models were presented by [40] for surveillance tasks at airports. Spatio-temporal features were automatically utilised by employing 3D convolutions in the convolutional layers with respect to spatial and temporal dimensions. The experimental results demonstrated superior performance for this method in comparison to other state-of-the-art methods.

In general, there has been much success with 2D and 3D CNN in e.g., image classification, object recognition, speech recognition and action recognition. Nonetheless, some issues still need to be considered such as the immense amount of image or video data needed for training purposes. Collecting and annotating large amounts of image or video data are quite exhausting and requires a substantial amount of time. Fortunately, the availability of rich and relatively large action recognition datasets has provided a great support for designing such models in terms of their training and evaluation. A factorised 3D-CNN was proposed by [170] for human action recognition. The 3D-CNN was factorised into a standard 2D-CNN for spatial information at the lower layers and a 1D-CNN for the temporal information at the higher layers. This factorisation was to reduce the number of learning parameters and consequently reduce the computational complexity. Two benchmark datasets were used to evalauate the proposed method: UCF101 and HMDB51. The results showed comparable performance with state-of-the-art methods. Another spatio-temporal 3D-CNN approach was proposed by [171] for human action recognition. The authors used four public datasets to evaluate the proposed method. The 3D-CNN achieved improved performance with spatio-temporal features compared to a 2D-CNN. The authors also found that a small filter size such as the one used in their method i.e., 3×3×3 was the best choice for spatio-temporal features. Overall, the experimental results demonstrated competitive performance for the proposed method with a linear classifier.

Some research works have combined supervised and unsupervised learning models for action recognition. A Slow Feature Analysis (SFA) based method has used by [172] to extract slowly varying features from an input in an unsupervised manner. These were combined with a 3D-CNN for action recognition. This work achieved competitive performance compared to state-of-the-art approaches. Three standard action recognition datasets were used: KTH [98], UCF sports [85] and Hollywood2 [99] datasets.

In [173], a hierarchical framework combining 3D CNN and hidden Markov model (HMM) was proposed. This was used to recognise and segment continuous actions simultaneously. 3D CNN was used to learn a powerful high level features directly from raw data, and use it to extract effective and robust action features. The statistical dependencies over adjacent sub-actions was then modeled by HMM to infer actions sequences. The KTH and Weizmann dataset were used to evaluate the proposed method. The experimental results showed improved performance of the proposed method over some state-of-the-art approaches.

**Figure 2:**
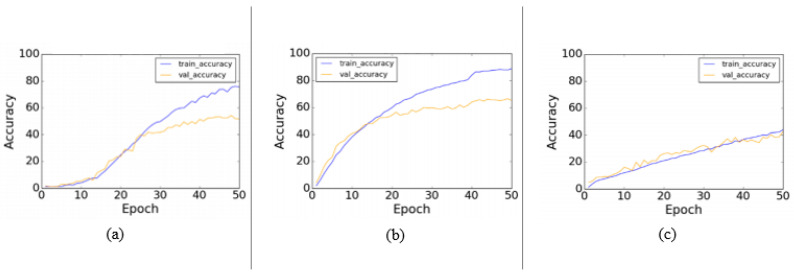
The performance of action recognition models as mentioned in [174]. Including: (**a**) Semi-CNN model based on VGG16 architecture (**b**) Semi-CNN model based on ResNet34 architecture (**c**) Semi-CNN model based on DenseNet121 architecture.

For efficient learning of spatio-temporal features in video action recognition, a hybrid CNN was introduced in [174] used a fusion convolutional architecture. 2D and 3D CNN was fused to present temporal encoding with fewer parameters. Three models are used to build the proposed model (semi-CNN) including: VGG-16, ResNets and DenseNets. The UCF-101 dataset was used in the evaluation to compare the performance of each model with its corresponding 3D models. Figure 2 shows the performance of the used models over 50 epochs.

Another way to model motion information in video was proposed by [39] for action recognition using Recurrent Neural Networks (RNN)s. CNN discriminative features were computed for each video frame and then they were fed into an RNN model. The key advantage of an RNN architecture is its ability to deal with sequential inputs as a single copy of the network is created for each sequence. In the RNN hidden layers, connections between neurons are found between each replica where the same weights are shared by each replica and with the others. The authors highlighted that local motion information can be obtained from video by optical flow through CNNs. On the other hand, global motion information can be modeled through the use of the RNN. RNN based supervised learning was used by [175] across five parts (right arm, left arm, right leg, left leg, trunk) of skeleton information. These were used as inputs to five separate sub-nets for action recognition. The outcomes of these sub-nets were then hierarchically fused to form the inputs to the higher layers. Thereafter, the final representation was fed into a single-layer perceptron to get the final decision. Three datasets were used to evaluate the proposed method including: MSR Action3D [74], Berkeley Multimodal Human Action (Berkeley Mhad) [176], and Motion Capture HDM05 [177] datasets. The results demonstrated state-of-the-art performance. However, RNN is not capable of processing very long sequences and it can not be stacked into very deep models. In addition, it lacks the capability of keeping track of long-term dependencies; which makes training of an RNN difficult.

New recurrent modules that improved long-range learning, Long Short-Term Memory (LSTM), has firstly proposed by [178]. LSTM units have hidden state augmented with nonlinear mechanisms, in which simple learned gating functions are utilised to enable state propagation with either no modification, update or reset. LSTMs have a significant impact on vision problems as these models are straightforward to fine-tune end-to-end. Moreover, LSTMs have the ability to deal with sequential data and are not limited to fixed length inputs or outputs. This helps to simply model a sequential data of varying lengths, such as text or video [179].

LSTMs have recently been introduced to be efficient to large-scale learning of speech recognition [180] and language translation models [181]. LSTM was also proposed for action recognition by [179]. A hybrid deep learning architecture was proposed using a long-term recurrent CNN (LRCN). Raw data and optical flow information were used as input to this unique system. The proposed methods were evaluated using a UCF101 dataset and showed an improvement in the performance in comparison with the baseline architecture.

Deep learning based approaches have achieved relatively high recognition performance. This is on the same level or better than hand-crafted features based methods. Some researchers have also proposed using multiple deep learning models alongside hand-crafted features to achieve even better results such as [32,117,182].

### 5.3. Multiple Modality Based Methods

A new insight is provided into human action recognition by using deep learning methods to extract action features from RGB, depth, and/or skeleton information. Different feature learning can be utilised [117,171,183] from deep networks such as appearance, optical flow, depth and/or skeleton sequences. It is very often that different modalities are provided with respect to the same dataset such as RGB, depth, and skeleton information or at least two of them. Therefore, a lot of research has been proposed to utilise combinations of different modalities or their hand-crafted features. They then merge them using fusion based strategies. A separate framework architecture is often employed for each modality; then, classification scores are often obtained for each one.

Some research has highlighted that significant improvements in performance of an action recognition system can be achieved by utilising hand-crafted features within CNN based deep learning models. A CNN model based on multiple sources of information was proposed by [184] to process spatially varying soft-gating. A fusion technique was then used to combine the multiple CNN models that were trained on various sources. A Stratified Pooling based CNN (SPCNN) was proposed by [185] to handle the issue of different feature levels of each frame in video data. To come up with video based features, the authors fine-tuned a pre-trained CNN model on target datasets. Frame-level features were extracted, then principal component analysis was used for dimensionality reduction. Stratified pooling of frame-level features was then used to convert them into video-level features, and finally fed them into an SVM classifier for classification. The method was evaluated on HMDB51 [27] and UCF101 [186] datasets. The experiments showed that the proposed method outperformed the state-of-the-art.

An extension of this two stream network approach was proposed in [117] using dense trajectories for more effective learning of motion information.

A general residual network architecture for human activity recognition was presented in [187] using cross-stream residual connections in the form of multiplicative interaction between appearance and motion streams. The motion information was exploited using stacked inputs of horizontal and vertical optical flow.

A fusion study was presented in [182] for human activity recognition using two streams of the pre-trained Visual Geometry Group (VGG) network model to compute spatio-temporal information combining RGB and stacked optical flow data. Various fusion mechanisms at different positions of the two streams were evaluated to determine the best possible recognition performance.

Some research studies have paid particular attention to auxiliary information which can improve the performance of action recognition. In some studies, audio has been combined with the video to detect the actions such as [188], where a combination of Hidden Markov Models (HMM) with audio were used to determine the actions. The main disadvantage of using audio recordings is the surrounding noise that can affect the results.

**Figure 3:**
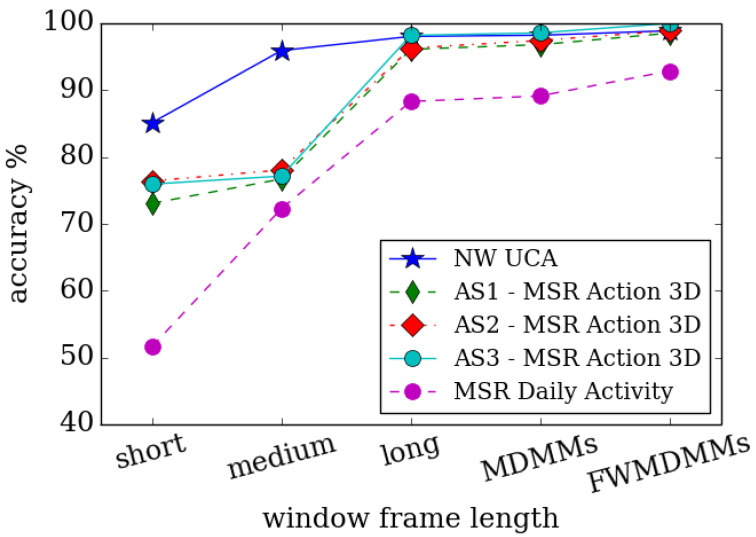
Action recognition accuracy versus different window frame lengths that was proposed in [189].

All of the above approaches suffer from a shortage of long-term temporal information. For example, the number of frames used in the optical flow stacking ranged between 7 and 15 frames, such as 7, 10, and 15 frames as used in [40,169,184], respectively. Often, people will perform the same action over different periods of time depending on many factors and particularly for different people. Consequently, multi-resolution hand-crafted features computed over different periods of time are used by [189] in order to avoid this problem. Furthermore, different weight phases are applied using a time-variant approach in the computation process of the DMMs to enable adaptation to different important regions of an action. Different fusion techniques are employed to merge spatial and motion information for best action recognition. Figure 3 illustrates the impact of different window frame lengths on the performance of action recognition systems.

### 5.4. Pose Estimation and Multi-View Action Recognition

Another considerable challenge in human action recognition is view variance. The same action can be viewed from different angles and thus looks excessively different. This issue was taken into account by [190]. Training data were generated by fitting a synthetic 3D human model to real motion information. Poses were then extracted from different view-points. A CNN based model was found to outperform a hand-crafted feature based approach for multi-view action recognition.

**Figure 4:**
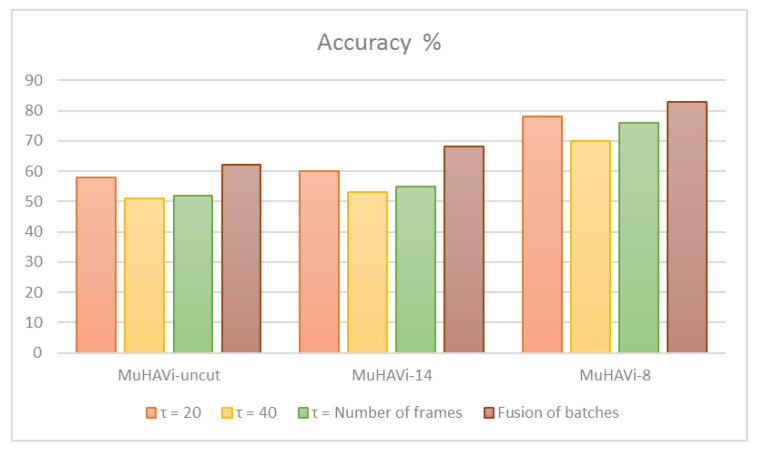
The accuracy variations with the number of frames and number of batches as mentioned in [192].

Dynamic image information was extracted by [191] from synthesised multi-view depth videos. Multi-view dynamic images were constructed from the synthesised data. A CNN model was then proposed to perform feature learning from the multi-view dynamic images. Multiple batches of motion history images (MB-MHIs) have been constructed by [192]. This information is then used to compute two descriptors by using: a deep residual network (ResNet) and histogram of oriented gradients (HOG). Later, an orthogonal matching pursuit approach was used to obtain the sparse codes of feature descriptions. A final view-invariant feature representation was formed and used to train SVM classifier for action recognition. MuHAVi-MAS [193] and MuHAVi-uncut [194] datasets are used to evaluate the proposed approach. Figure 4 illustrates the accuracy variations of the recognition model over different components.

A CNN model obtained from ImageNet was used by [195] to learn from multi-view DMM features for action recognition when video was projected onto different view-points within the 3D space. Different temporal scales were then used from the synthesised data to constitute a range of spatio-temporal pattern for each action. Finally, three fine-tuned models were employed independently for each DMM map. However, some actions including object interactions can be very difficult to be recognise from the raw depth data alone. This helps to justify the inclusion of RGB data for the recognition of such actions.

In [196], Multi-View Regional Adaptive Multi temporal-resolution DMMs (MV-RAMDMM) and Multi temporal-resolution RGB information is learnt with multiple 3D-CNNs stream for action recognition. The Adaptive Multi-resolution DMM is applied across multiple views to extract view and time invariant action information. It is adapted based on human movement to be used eventually in the deep learning model for action recognition. In addition, multi temporal raw appearance information is used to exploit various spatio-temporal features of the RGB scenes. This helps to capture more specific information which might be difficult to obtain purely from depth sequences. For instance, object-interaction information is more apparent in RGB space.

In a different way, semantic features based on pose can be seen to be very important cues that can describe the category of an action. Human joint information was utilised by [197] to compute the temporal variation between joints during actions. Time-variant functions were used to confirm the pose related with each action and considered for feature extraction. The feature representation for action recognition was constructed using the temporal variation of values associated with these time functions. Then, CNNs were trained to recognize human actions from the local patterns in the feature representation. The Berkeley MHAD dataset [176] was used to evaluate the proposed method and the results demonstrated the effectiveness of this approach. Similar to [197], a Pose-based Convolutional Neural Network descriptor (P-CNN) for action recognition was proposed by [198]. Descriptor aggregated motion and appearance information were used with respect to tracks of human body parts. This utilised skeleton information along with RGB raw data. JHMDB [199] and MPII [200] cooking datasets were used to evaluate the proposed method. However, it can be difficult to accurately capture skeleton information of a person in different environment conditions. This might be due to the need of accurate body-parts detection to precisely estimate skeleton information.

Some common datasets of human action recognition are introduced in Table 2. In addition, an extensive comparison between deep learning based models and hand-crafted based models are presented in Table 3 for human action recognition.

Furthermore, some recent works based on deep learning models for human action recognition are included in Table 4.

## 6. Conclusions

In this paper, we have presented human action recognition methods and introduced a comprehensive overview of recent approaches to human action recognition research. This included a hand-crafted representation based method, deep learning based methods, human–object interaction and multiview action recognition. The conclusions of this study on human action recognition can focus on the following:

- data selection: suitable data to capture the action may help to improve performance of action recognition.
- approach of recognition: deep learning based methods achieved superior performance.
- multiple-modal: current research highlighted that multi-modal fusion can efficiently improve the performance.

This paper has presented the most relevant and outstanding computer vision based methods for human action recognition. A variety of hand-crafted methods and deep learning models have been summarised along with various advantages and disadvantages for each approach. Hand-crafted feature based methods are categorised into holistic and local feature based methods. Holistic feature based methods have been summarised along with their limitations. These methods assume a static background. In other words, the camera must be stable and videos are supposed to have been captured in a constrained condition for a holistic representation. Otherwise, these methods need extra pre-processing steps such as people detection to be able to recognise human actions. This is particularly true in the presence of cluttered or a complex background or if the camera moves whilst action sequences are captured. Next, local feature based methods and different types of descriptors were also described in this paper. It is shown that local feature based methods more often achieve state-of-the-art results compared to other approaches. In addition, such kinds of methods require reduced computational complexity to recognise human actions compared to more complicated models. The main advantage of local feature based methods is their flexibility. They can be applied to video data without complex requirements such as human localisation or body parts detection, which is not feasible for many types of videos. However, in some cases, it is very difficult to address action variations using local representation based methods, which, in turn, fails to precisely recognise human actions. Therefore, using hand-crafted representations by taking advantage of combining both local and holistic based methods may help. Different issues are tackled benefiting from shape and motion information, and local feature representation of an action. This information alongside local representation strategies are considered as the key roles for recognising different actions and improving the performance of the recognition system.

A new direction has been proposed to enhance the action recognition performance using deep learning technology. Deep learning is summarised in this paper and classified into two categories including: supervised and unsupervised models. However, supervised models are considered in this work due to their vast ability and high effectiveness in implementing recognition systems. It has achieved competitive performance in comparison with traditional approaches in many applications of computer vision. The most important characteristic of deep learning models is the ability to learn features from raw data. This has somewhat reduced the need for hand-crafted feature detectors and descriptors.

One of the most popular supervised models is the Convolution Neural Network (CNN), which is currently being used in most of the existing deep learning based methods. However, deep learning based methods still have some limitations that need to be considered. One of these limitations is the need for huge amounts of data for training the models. In addition, there is a high-complexity hardware requirement to enable computation in a plausible amount of time. Therefore, transfer learning approaches are adopted in different works to benefit from pre-trained models to speed up the training processes. This also helps to improve the performance of the action recognition system with reasonable hardware requirements.

Two common types of deep learning techniques were used for either spatial or spatio-temporal feature extraction and representation. This can include CNN, 3D CNN, LSTM, etc. Some research has highlighted that significant improvements in performance of an action recognition system can be achieved by utilising multi-modalities structure based methods. This could include RGB sequences, hand-designed features, depth sequences and/or skeleton sequences.

Many researchers have highlighted the importance of temporal information that can be exploited to provide more discriminative features for action recognition. This information was processed early with an independent 2D-CNN stream.

Spatio-temporal features have also been learnt directly with the use of 3D-CNN or LSTM models. These have been summarised in this review in which temporal domain has been considered in the learning process. Multi-modalities structure may add great improvements to the recognition system within a deep learning model. Toward this aim, different action recognition systems were presented within different temporal batches involving a deep learning model. 

## Figures and Tables

**Table 1:** State-of-the-art methods of traditional hand-crafted representations with different datasets for human action recognition.

Paper	Year	Method	Dataset	Accuracy
[136]	2009	Space-time volumes	KTH	89.4
[101]	2011	Dense trajectory	KTH	95
[137]	2011	Space-time volumes	KTH	94.5
			UCF sports	91.30
[138]	2011	Shape-motion	Weizmann	100
[139]	2011	LBP	Weizmann	100
[140]	2012	bag-of-visual-words	HDMB-51	29.2
[141]	2012	Trajectory	HDMB-51	40.7
[142]	2012	HOJ3D + LDA	MSR Action 3D	96.20
[143]	2013	Features (Pose-based)	UCF sports	90
			MSR Action 3D	90.22
[144]	2013	3D Pose	MSR Action 3D	91.7
[145]	2013	Shape Features	Weizmann	92.8
[111]	2013	Dense trajectory	HDMB-51	57.2
[146]	2014	Shape-motion	Weizmann	95.56
			KTH	94.49
[147]	2014	EigenJoints + AME + NBNN	MSR Action 3D	95.80
[148]	2014	Features (FV + SFV)	HDMB-51	66.79
			Youtube action	93.38
[149]	2014	Dissimilarity and sparse representation	UPCV Action dataset	89.25
[150]	2014	Shape features	IXMAS	89.0
[151]	2016	Trajectory	MSR Action 3D	89
[152]	2016	Shape Features	Weizmann	100
[153]	2016	Shape features	IXMAS	89.75
[154]	2016	LBP	IXMAS	80.55
[155]	2016	Motion features	IXMAS	83.03
[64]	2017	MHI	MuHAVi	86.93
[156]	2017	spatio-temporal+HMM	MSR Action 3D	93.3
			MSR Daily	94.1
[157]	2018	Joints + KE Descriptor	MSR Action 3D	96.2

**Table 2:** Common dataset of human action recognition.

Datasets	RGB	Depth	Skeleton	Samples	Classes
*KTH [98]*	**✓**	**✗**	**✗**	1707	12
*Weizmann [201]*	**✓**	**✗**	**✗**	4500	10
*Hollywood2 [99]*	**✓**	**✗**	**✗**	1707	12
*HMDB51 [27]*	**✓**	**✗**	**✗**	6766	51
*Olympic Sports [202]*	**✓**	**✗**	**✗**	783	16
*UCF50 [203]*	**✓**	**✗**	**✗**	6618	50
*UCF101 [186]*	**✓**	**✗**	**✗**	13,320	101
*MSR-Action3D [74]*	**✗**	**✓**	**✓**	567	20
*MSR-Daily Activity [107]*	**✓**	**✓**	**✓**	320	16
*Northwestern-UCLA [204]*	**✓**	**✓**	**✓**	1475	10
*Berkeley-MHAD [205]*	**✓**	**✓**	**✓**	861	27
*UTD-MHAD [205]*	**✓**	**✓**	**✓**	861	27
*RGBD-HuDaAct [206]*	**✓**	**✓**	**✗**	1189	13
*NTU RGB+D [207]*	**✓**	**✓**	**✓**	56,880	60

**Table 3:** Comparison of deep learning based models and hand-crafted based models for human action recognition [208,209,210,211].

Characteristics	Deep Learning Based Models	Hand-Crafted Feature Based Models
*Feature extraction and Representation*	Ability to learn features directly from raw data	Pre-process algorithms and /or detectors are needed to discover the most efficient patterns to improve recognition accuracy.
*Generalisation and Diversity*	Automatically extract spatial, temporal and scale, transition invariant features from raw data	Use feature selection and dimensionality reduction methods which are not very generalisable.
*Data preparation*	Data pre-processing and normalisation is not mandatory in deep learning based models to achieve high performance	Usually require comprehensive data pre-processing and normalisation to achieve significant performance.
*Inter-class and Intra-class*	Hierarchical and translational invariant features are obtained from such models to solve this problem	Inefficient in managing such kind of problems.
*Training and Computation time*	Huge amount of data required for training purposes to avoid over-fitting and high computation powerful system with Graphical Processing Unit (GPU) to speed up training	Require less data for training purposes with less computation time and memory usage.

**Table 4:** State-of-the-art methods of deep learning based models with different datasets for human action recognition.

Paper	Year	Method	Class of Architecture	Dataset	Accuracy
[212]	2012	ASD features	SFA	KTH	93.5
[40]	2013	Spatio-temporal	3D CNN	KTH	90.2
[163]	2014	STIP features	Sparse auto-encoder	KTH	96.6
[32]	2014	Two-stream	CNN	HDMB-51	59.4
[172]	2014	DL-SFA	SFA	Hollywood2	48.1
[32]	2014	Two-stream	CNN	UCF-101	88.0
[213]	2015	convolutional temporal feature	CNN-LSTM	UCF-101	88.6
[117]	2015	TDD Descriptor	CNN	UCF-101	91.5
[170]	2015	Spatio-Temporal	CNN	UCF-101	88.1
[171]	2015	Spatio-temporal	3D CNN	UCF-101	90.4
[175]	2015	Hierarchical model	RNN	MSR Action3D	94.49
[214]	2015	Differential	RNN	MSR Action3D	92.03
[215]	2015	static and motion features	CNN	UCF Sports	91.9
[117]	2015	TDD Descriptor	CNN	HDMB-51	65.9
[170]	2015	Spatio-Temporal	CNN	HDMB-51	59.1
[216]	2016	Spatio-temporal	LSTM-CNN	HDMB-51	55.3
[184]	2016	Deep Network	CNN	UCF-101	89.1
[216]	2016	Spatio-temporal	LSTM-CNN	UCF-101	86.9
[184]	2016	Deep model	CNN	HDMB-51	54.9
[173]	2016	3D CNN + HMM	CNN	KTH	89.20
[179]	2016	LRCN	CNN + LSTM	UCF-101	82.34
[185]	2017	SP-CNN	CNN	HDMB-51	74.7
[217]	2017	Rank pooling	CNN	HDMB-51	65.8
[217]	2017	Rank pooling	CNN	Hollywood2	75.2
[185]	2017	SP-CNN	CNN	UCF-101	91.6
[218]	2018	DynamicMaps	CNN	NTU RGB+D	87.08
[219]	2018	Cooperative model	CNN	NTU RGB+D	86.42
[191]	2019	Depth Dynamic Images	CNN	UWA3DII	68.10
[189]	2019	FWMDMM	CNN	MSR Daily Activity	92.90
			CNN	NUCLA	69.10
[192]	2020	MB-MHI	ResNet	MUHaVi	83.8
[196]	2020	MV-RAMDMM	3DCNN	MSR Daily Activity	87.50
			3DCNN	NUCLA	86.20
[174]	2020	Semi-CNN	ResNet	UCF-101	89.00
		Semi-CNN	VGG-16	UCF-101	82.58
		Semi-CNN	DenseNet	UCF-101	77.72
